# Supplementary material for: Gelatin-Based Electrospun Nanofibers Cross-Linked Using Horseradish Peroxidase for Plasmid DNA Delivery
Source: Biomolecules. 2022 Nov 4;12(11):1638. doi: 10.3390/biom12111638 (PMC9687400; doi:10.3390/biom12111638)
Supplement: Supplementary file 1 [file biomolecules-12-01638-s001.zip › biomolecules-1989147-SI/Supplementary Material.pdf]

Supplementary

Gelatin-based Electrospun Nanofibers Cross-linked using Horseradish Peroxidase for Plasmid DNA Delivery

Kotoko Furuno[a], Keiichiro Suzuki[a, b, c] and Shinji Sakai[a]

[a]Department of Materials Engineering Science, Graduate School of Engineering Science, Osaka University, 1-3 Machikaneyama-cho, Toyonaka, Osaka 560-8531, Japan

[b]Institute for Advanced Co-Creation Studies, Osaka University, 1-3 Machikaneyama-cho, Toyonaka, Osaka 560-8531, Japan

[c]Graduate School of Frontier Bioscience, Osaka University, 1-3 Yamadaoka, Suita, Osaka 565-0871, Japan

Number of pages: 2

Number of figures: 2

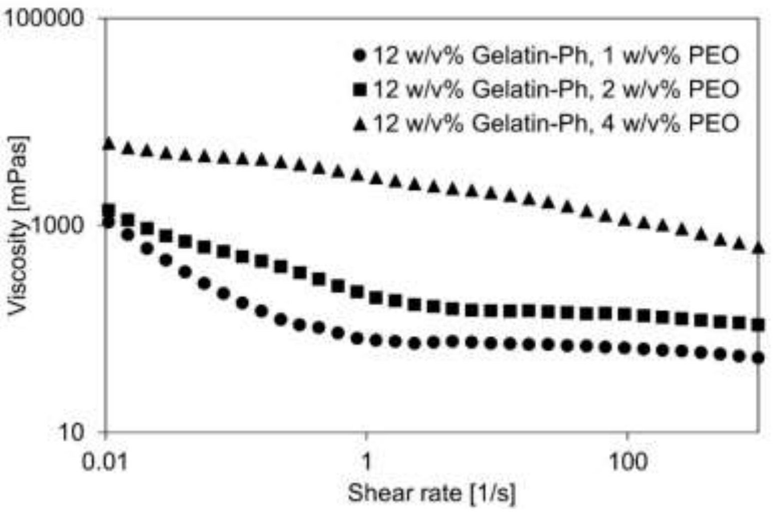

Figure S1 Viscosity of the solutions. The solutions contained 12 w/v% Gelatin-Ph and 2, 4, or 8 w/v% PEO.

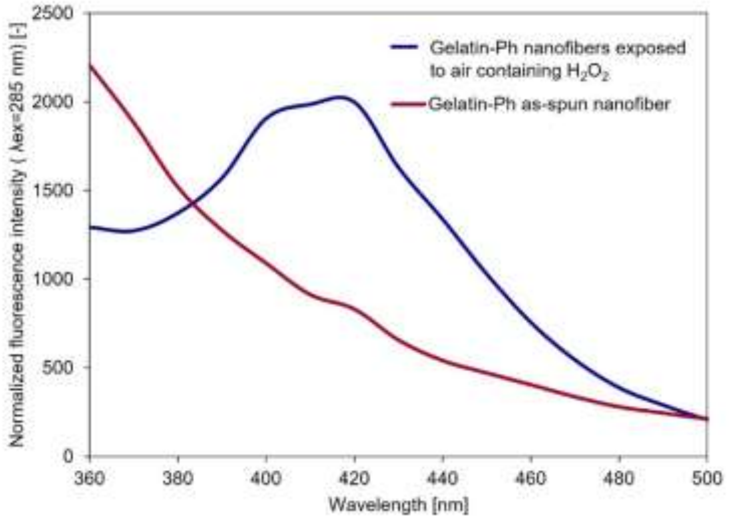

Figure S2 Fluorescence intensity of Gelatin-Ph nanofibers exposed to air containing H_2O_2 and Gelatin-Ph as-spun nanofibers. The fluorescence attributed to dityrosine (400–420 nm) was monitored with excitation wavelength of 285 nm.
